# Knowledge, Attitude and Decision-making of Nurses in the Resuscitation Team towards Terminating Resuscitation and Do-not-Resuscitate Order

**DOI:** 10.4314/ejhs.v32i2.22

**Published:** 2022-03

**Authors:** Afshin Goodarzi, Efat Sadeghian, Keivan Babaei, Masoud Khodaveisi

**Affiliations:** 1 Ph.D. Student in Nursing, School of Nursing and Midwifery, Hamadan University of Medical Sciences, Hamadan, Iran; 2 Department of Emergency Medicine, School of Para medicine, Kermanshah University of Medical Sciences, Kermanshah, Iran; 3 Chronic Diseases (Home Care) Research Center, Department of Nursing, Hamadan University of Medical Sciences, Hamadan, Iran; 4 Chronic Diseases (Home Care) Research Center, Department of Community Health Nursing, Hamadan University of Medical Sciences, Hamadan, Iran

**Keywords:** Cardiopulmonary resuscitation, DNR order, Ethics, Resuscitation Orders

## Abstract

**Background:**

Making appropriate decisions for cardiopulmonary resuscitation (CPR) is very challenging for healthcare providers. This study aimed to evaluate knowledge, attitude, and decision making about do-not-resuscitate (DNR) and termination of resuscitation (ToR) among nurses in the resuscitation team.

**Methods:**

This descriptive cross-sectional study was conducted in April-September 2020. Participants were 128 nurses from the CPR teams of two hospitals in Kermanshah and Hamedan, Iran. A valid and reliable researcher-made instrument was used for data collection. Data were analyzed using the Chi-square, Fisher's exact, and Mann-Whitney U tests, the Spearman's correlation analysis, and the logistic and rank regression analyses.

**Results:**

Only 22.7% and 37.5% of participants had adequate knowledge about ToR and DNR. The significant predictor of DNR and ToR knowledge was educational level and the significant predictors of decision making for CPR were educational level, gender, and history of receiving CPR-related education (P<0.05). When facing a cardiac arrest and indication of DNR or ToR, 12.5% of participants reported that they would not start CPR, 21.5% of them reported that they would terminate CPR, and 14.8% of them reported that they would perform slow code. The DNR decision had significant relationship with educational level, DNR knowledge, and ToR knowledge (P< 0.05), while the ToR decision had significant relationship with educational level and ToR knowledge (P<0.05).

**Conclusion:**

Nurses' limited DNR and ToR knowledge and physicians' conflicting orders and documentation can cause ethical challenges for nurses. Clear guidelines for DNR orders or TOR is necessary for nurses, in order to prevent any potential confusion, legal or psychosocial issues and concerns surrounding CPR and improve their involvement in CPR decision making process.

## Introduction

Despite the significant decrease in cardiovascular deaths in high-income countries in the past twenty years, cardiac arrest is still a leading cause of death worldwide (1–3). The annual rate of cardiac arrest among adults is 357900 cases in the United States (4) and 2.7–11.7 cases per 1000 hospitalizations in Asian countries (5–7). Inappropriate management of cardiac arrest results in death, while cardiopulmonary resuscitation (CPR) can prevent death in some patients (8). Survival-to-discharge rate after successful CPR is 18% in the United Kingdom and 28% in Scandinavian countries (9, 10). Although modern CPR has a history of around seventy years (11–15) and its guidelines are updated every five years (16), there are serious challenges in determining the criteria for termination of resuscitation (ToR) (17), particularly in developing countries (18). The three types of withholding CPR include ToR in unsuccessful CPR, do not resuscitate (DNR) in futile CPR, and DNR based on patient's previous request (19, 20). Healthcare providers are usually believed that CPR is not appropriate for every patient. Frequent performance of CPR on terminally ill patients raised concerns that these resuscitations were often utilized inappropriately. This lead to the emergence of DNR policy to identify patients who would not benefit from CPR (21, 22).

CPR in Islamic cultures has more complex challenges. According to the principles of Islam, each moment of human life has great value and hence, planned death is never accepted in Islam. On the other hand, death in Divine religions is considered as a stage of life rather than the end of life. Therefore, prolongation of the process of death is not justified in these religions (23–27). Accordingly, specific culturally appropriate CPR guidelines have been developed in some Muslim countries based on the immediate ethical principles (28, 29). In Iran, some factors related to DNR or ToR have been identified (19, 20, 27, 30); however, there is no national guideline in this area.

Lack of clear guidelines for DNR or ToR affects CPR team members' decisions, causes ambiguities and conflicts in CPR, makes physicians not document DNR or ToR orders in patients' medical records due to their fear over its legal consequences, and leads to futile interventions or slow code, pangs of conscience, and wastage of time and resources (31–33). A study in Iran found that 76.1% of physicians verbally and informally ordered DNR and 59.4% of them issued alternative orders such as slow code (32). Slow code by prehospital emergency medical services staff was also reported in 27% cases of cardiac arrest (20). Although decisions about ToR or DNR are mainly made by physicians, other CPR team members, including nurses, may affect these decisions, particularly in teaching hospitals where medical residents and assistants with limited CPR experience lead CPR teams (34–36). Therefore, nurses' CPR knowledge is of great importance in decision making about DNR and ToR. Nonetheless, studies reported that nurses in CPR teams have limited CPR knowledge. A study showed that only 5% of nurses knew the determining factors of ToR (33). Another study found that the level of nurses' knowledge about the ethical codes of CPR was unacceptable (37). Several studies also reported that healthcare providers' educational level, family members' insistence, and patients' underlying conditions had significant effects on CPR-related decisions (32, 33, 38, 39). Also, other factors such as religion, culture, race and geographical location can potentially affect the attitude of healthcare providers toward a DNR order or ToR (39).

Despite the wealth of studies into the different aspects of CPR, there are limited studies into its ethical codes and decision making about DNR and ToR. Moreover, most studies in this area were conducted on physicians and hence, there are limited data on nurses' decision making in CPR. The present study was conducted to address this gap. The aim of the study was to evaluate DNR and ToR knowledge, attitude, and decision making among nurses in the resuscitation team.

## Methods

This descriptive cross-sectional study was conducted in April–September 2020. Study setting was the emergency wards of Imam Reza (PBUH) hospital, Kermanshah, Iran, and Besat hospital, Hamedan, Iran. Study population consisted of all 164 nurses in CPR teams in these two hospitals. In total, 128 eligible nurses were recruited through purposive sampling. Inclusion criteria were bachelor's degree or higher in nursing and membership in CPR team for at least one year. The target population in the present study to make tools were specialists, nursing professors, and experienced members of the resuscitation code in the emergency department at Hamadan and Kermanshah universities of medical sciences from which 10 people were purposefully selected. In this regard, it was tried that the selected samples are completely representative of the target community. First, in a review study, the items of the instrument were generated using the latest international CPR guidelines and the existing literature on CPR (20, 30–34, 38–46). In the second stage, all the documents obtained from the review study and the items extracted based on them were provided to all selected experts from the target community. This step was performed by the Delphi method in three parts (round trip) and finally, the tool structure was designed by selecting the appropriate items. The final instrument had three main dimensions. The first dimension was related to ToR knowledge and included twelve Yes/No questions. Positive response to item 6 and negative response to the remaining items were interpreted as having ToR knowledge. Item 6 was on “cardiac asystole for more than twenty minutes despite the management of hyperkalemia”. The second dimension of the instrument was related to DNR knowledge with nine Yes/No items. Positive response to item 6 and negative response to the remaining items were interpreted as having necessary DNR knowledge. Item 6 was on “the symptoms of irreversible death including livor mortis and rigor mortis”. The third dimension of the instrument included 24 items on CPRrelated attitudes. The first three items of this dimension were on nurses' perceived competence and decision-making skills for CPR management. The next fifteen items were on nurses' attitude about the effects of factors irrelevant to DNR and ToR orders and the remaining items were on nurses' attitude about physicians' decision making for DNR and ToR and their documentation.

The validity and reliability of the study instrument were assessed through qualitative and quantitative face validity assessments, quantitative content validity assessment by calculating content validity index and ratio, construct and convergent validity assessments, and internal consistency assessment. The scale-level content validity index of the instrument was 0.8, indicating acceptable content validity (47). The Cronbach's alpha of the instrument was 0.816 with the data obtained from fifteen participants and 0.714 with the data obtained from all participants. Cronbach's alpha values greater than 0.7 show acceptable reliability and convergent validity (48).

**Sample size estimation**: With a statistical power of 90%, and a type I error of 5%, Cochran's sample size calculation formula revealed that 116 nurses were needed. Nonetheless, considering a probable attrition rate of 10%, sample size was increased to 128.

**Statistical analysis**: Data were analyzed at a significance level of less than 0.05 using the SPSS software (v. 20.0). Normality of the study variables was tested using the Kolmogorov-Smirnov test. The Chi-square, Fisher's exact, and Mann-Whitney *U* tests as well as the Spearman's correlation analysis were used to analyze the relationships of ToR knowledge, DNR knowledge, and CPR-related decision making with participants' demographic characteristics. The logistic and the rank regression analyses were also used to determine the predictors of DNR knowledge, ToR knowledge, and CPR-related decision making. Variables with significant relationships with DNR knowledge, ToR knowledge, and CPR-related decision making at a significance level of less than 0.1 were entered into the regression models. P-value <0.05 was considered statistically significant.

**Ethical considerations**: The Institutional Review Board and the Ethics Committee of Hamedan University of Medical Sciences, Hamedan, Iran, approved this study (codes: 9903061375 and IR.UMSHA.REC.1399.205). Study instrument was anonymous and participants were informed about the study aim. Verbal informed consent was obtained from all participants.

## Results

In total, 128 nurses participated in the study. The mean of their age and work experience was 34.63±18.8 and 10.32±7.80 years, respectively. Most of them were female (68.8%), had bachelor's degree (80.5%), and reported participation in continuing education programs on the ethical codes of CPR (53.1%). Only 22.7% of participants had ToR knowledge and 37.5% of them had DNR knowledge, while the other participants erroneously reported old age, malignancy, treatment-resistant cardiac dysrhythmia, or multiple organ failure as the indications of DNR or ToR. DNR knowledge had significant relationship with age, educational level, and work experience, while ToR knowledge had significant relationship with educational level (*P*<0.05; Table 1).

**Table 1:** The relationships of participants' demographic characteristics with their DNR and ToR knowledge

Variables		Mean±SD or N (%)	DNR Knowledge N (% or Mean Rank)	P value	TOR Knowledge N (% or Mean Rank)	P value
**Age (years)**	23–54	34.63±18.8	48 (79.79)	<0.001	29 (72.21)	0.203
**Work experience (yrs)**	1–30	10.32±7.80	48 (78.85)	0.001	29 (70.03)	0.360
**Gender**	Male	40 (31.2)	16 (40)	0.694	8 (20)	0.628
	Female	88 (68.8)	32 (36.36)		21 (23.86)	
**Marital status**	Single	56 (43.8)	16 (28)	0.066	12 (21)	0.77
	Married	72 (56.3)	32 (44)		17 (24)	
**Educational level**	Bachelor's	103 (80.5)	29 (28)	<0.001	14 (14)	<0.001
	Master's	25 (19.5)	19 (76)		15 (60)	
**History of receiving**	Yes	68 (53.1)	26 (38.23)	0.85	15 (22.05)	0.864
**CPR-related education**	No	60 (46.9)	22 (36.66)		14 (23.33)	

When facing a cardiac arrest which has the indication of DNR or ToR, 12.5% of participants reported that they would not start CPR, 21.5% of them reported that they would terminate CPR, and 14.8% of them reported that they would perform slow code. The DNR decision had significant relationship with educational level, DNR knowledge, and ToR knowledge (*P*< 0.05), while the ToR decision had significant relationship with educational level and ToR knowledge (P < 0.05). Moreover, the decision to perform slow code had significant relationship with gender and history of receiving CPR-related education (P < 0.05) (Table 2).

**Table 2:** The relationships of participants' demographic characteristics, DNR knowledge, and ToR knowledge with their CPR-related decisions

Variables		Decision on DNR N (CC or Mean Rank)	*P*- value	Decision on ToR N (CC or Mean Rank)	*P*- value	Decision on slow code N (CC or Mean Rank)	*P*- value
**Age (Years)**	23–54	128 (0.083)	0.350	128 (0.169)	0.102	128 (0.150)	0.101
**Work** **experience (Yrs)**	1–30	128 (0.105)	0.238	128 (0.153)	0.124	128 (0.144)	0.104
**Marital status**	Single	56 (66.56)	0.522	56 (60.04)	0.199	56 (62.97)	0.639
	Married	72 (62.90)		72 (6797)		72 (65.69)	
**Educational** **level**	Bachelor's	103 (61.92)	0.046	103 (58.24)	<0.001	103 (65.86)	0.336
	Master's	25 (75.12)		25 (90.30)		25 (58.90)	
**History of** **receiving CPR**- **related** **education**	Yes	68 (66.72)	0.406	68 (69.48)	0.083	68 (58.66)	0.030
	No	60 (61.98)		60 (58.86)		60 (71.12)	
**Gender**	Male	40 (65.69)	0.778	40 (68.11)	0.426	40 (73.59)	0.033
	Female	78 (63.96)		88 (62.86)		88 (60.37)	
**DNR** **Knowledge**	Yes	48 (73.83)	0.011	48 (66.28)	0.652	48 (61.97)	0.494
	No	80 (58.90)		80 (63.43)		80 (66.02)	
**ToR Knowledge**	Yes	29 (75.41)	0.038	29 (81.12)	0.003	29 (67.21)	0.556
	No	99 (61.30)		99 (59.63)		99 (63.71)	

CC- Correction cooficient

The logistic regression analysis revealed that educational level was the only significant predictor of DNR and ToR knowledge and predicted 15%–23% of the total variance (Table 3). Moreover, the rank regression analysis showed that male gender and no history of receiving CPR-related education were the significant predictors of the decision to perform slow code, while educational level was the only predictor of the ToR decision. These variables predicted 64%–85% of the total variance (Table 4).

**Table 3:** The predictors of DNR knowledge and ToR knowledge

Dependent variable	Independent variable	B	S.E.	Wald	df	Sig.	Exp (B)
DNR knowledge	Age	–.055	0.076	0.537	1	0.464	0.946
	Marital status	–.425	0.450	0.891	1	0.345	0.654
	Work experience	0.017	0.079	0.048	1	0.826	1.017
	Educational level	1.863	0.542	11.820	1	0.001	6.440
	Constant	1.032	1.992	0.269	1	0.604	2.808
ToR knowledge	Educational level	2.255	0.499	20.395	1	0.000	9.536
	Constant	0-.405	0.408	0.986	1	0.321	0.667

**Table 4:** The predictors of DNR decision, ToR decision, and slow code decision

Dependent variable	Independent variable	Estimate	Std. Error	Wald	df	Sig.	95% confidence interval	
							Lower Bound	Upper Bound
DNR decision	DNR knowledge	0.696	0.421	2.741	1	0.098	–0.128	1.521
	Termination knowledge	0.350	0.483	0.526	1	0.468	–0.596	1.296
	Educational level	0.224	0.490	0.208	1	0.648	0.737	1.184
		0^a^	.	.	0	.	.	.
ToR decision	Educational level	1.392	0.460	9.181	1	0.002	0.492	2.293
	Get training	0.524	0.341	2.362	1	0.124	–0.144	1.193
	Termination knowledge	0.735	0.431	2.903	1	0.088	–0.111	1.580
		0^a^	.	.	0	.	.	.
Slow code decision	Get training	–0.812	0.360	5.080	1	0.024	–1.518	-0.106
	Gender	0.866	0.375	5.342	1	0.021	0.132	1.600
		0^a^	.	.	0	.	.	.

According to the participants, malignancy and age were the most prevalent factors which encouraged CPR staff decide on DNR (18.8% vs. 16.4%), decide on ToR (23.4% vs. 22.7%), and decide on slow code (22.7% vs. 25%). They also reported unwitnessed cardiac arrest and absent pupil reflex as factors with the least effects on early ToR. They also reported unwitnessed cardiac arrest and companions' insistence on DNR as factors with the least effects on performing slow code.

Participants noted that medical orders respecting DNR and early ToR are never documented in patients' medical records in respectively 71.9% and 61.7% of cases. Moreover, 74.2% of them believed that physicians verbally order DNR or early ToR but document complete CPR in patients' medical records.

## Discussion

This study evaluated DNR and ToR knowledge, attitude, and decision making among nurses in the CPR team. Findings showed that respectively 22.7% and 37.5% of participants had ToR and DNR knowledge, and 53.1% of them had received education about the ethical codes of CPR. Most participants chose irrelevant factors as determining factors of DNR and ToR. In comparison with other CPR-related skills (45), these findings show that nurses in CPR teams have significantly lower knowledge about the ethical codes of CPR. A former study also reported that only 5% of nurses had adequate ToR knowledge (33). Another study found that the level of nurses' and emergency medical services staff's knowledge about the legal issues and the ethical codes of CPR was unacceptable (37). A study in Saudi Arabia revealed a lack of familiarity with DNR's policies and understanding when it comes to treating DNR-labeled patients among medical interns and residents (49).

Contrarily, a study on physicians showed that 96.4% of them had adequate DNR knowledge (46). These findings denote that despite great attention to the education of CPR skills to nurses, limited attention has been paid to education about the ethical codes of CPR probably due to the fact that healthcare authorities consider physicians responsible for making DNR or ToR decisions. However, it is worthy to note that in the absence of physicians experienced in CPR management, particularly in teaching hospitals, nurses need to actively participate in joint decision making for CPR (34–36). Therefore, it is needed to incorporate courses on CPR and its ethical issues in the formal curriculum of nursing and also in in-service continuing education programs.

Study findings also showed that despite the significant relationship of DNR and ToR knowledge with age, work experience, and educational level, the only significant predictor of DNR and ToR knowledge was educational level. In line with this finding, a former study reported educational level as a significant factor contributing to the level of theoretical knowledge about CPR (50). However, some studies reported contradictory results (37, 51). This contradiction is attributable to the differences in the content of CPR-related educational programs in different educational settings. Further studies are needed to produce more reliable evidence in this area.

Our findings also indicated that when facing a cardiac arrest which has the indication of DNR or ToR, only 12.5% of participants reported DNR, 21.5% of them reported ToR, and 14.8% of them reported performing slow code. Two former studies also reported the same findings (33, 41). Another study also showed that 59.4% of nurses performed slow code instead of DNR (32). These findings denote nurses' limited perceived competence in making appropriate DNR or ToR decisions. Lack of clear DNR and ToR guidelines and nurses' concerns over the legal consequences of inappropriate DNR or ToR can also result in their decision on slow code (31–33). Therefore, developing clear DNR and ToR guidelines based on the existing international CPR guidelines and the Islamic principles of ethical practice as well as effective in-service continuing education are recommended to improve nurses' competence in decision making for CPR. However, the results of a study in Iran showed that most of the nurses had a negative attitude towards many aspects of DNR orders. This negative attitude may be one of the barriers to legalized DNR orders in the country (52). Another study among Iranian nurses indicated that generally, nurses have a negative attitudes toward DNR order. However, nurses with more than 15 years of work experience had more positive attitude towards this order (53). Nonetheless, the results of a study in Turkey revealed that the most of the physicians and nurses had a positive view about DNR order and request that the DNR order to be legally implemented (54). Additionally, in another study in Turkey showed that DNR order prohibition was associated with increased the futile CPR attempts (55).

We also found that while DNR knowledge, ToR knowledge, and educational level had significant relationships with nurses' DNR and ToR decisions, educational level was the only significant predictor of ToR decision. Moreover, gender and history of receiving CPR-related education were the only significant predictors of the decision to perform slow code. In other words, male nurses and those who had not received education were more likely to decide on performing slow code. A former study reported that physicians compared with nurses and more experienced staff compared with less experienced staff felt more competent in deciding on ToR (33). Another study found that compared with staff with lower educational level, physicians and nurses were more likely to decide on DNR (38). Higher educational level, which was associated with DNR and ToR knowledge in the present study, can empower healthcare providers to make appropriate decisions (38, 39). A study by Fallahi et al. in Iran showed that nurses and physicians' had different viewpoints about decision making of DNR. This issue highlights needing for a guideline that clearly states the responsibilities of each CPR team members in this process (56).

A study among Jordanian critical care nurses indicated that the most of them believed that the patient's family should be involved in DNR decision making (57). Knowledge and attitude toward DNR among patients and their relatives may be helpful to healthcare providers to make better decisions that are legally and ethically acceptable for the patients and their family (58).

In this regard, the results of a study confirmed that there is a lack of knowledge regarding DNR among patients and their relatives (59).

Study findings also revealed malignancy and old age of patients as the most prevalent irrelevant factors which encouraged nurses to decide on DNR, ToR, or slow code. Two former studies also reported patients' underlying conditions as a significant factor for deciding on DNR (32, 33). Similarly, several studies reported age and absent pupil reflex as factors contributing to ToR (34, 35, 40, 60, 61). However, international CPR guidelines and national regulations highlight that DNR and ToR are indicated only in case of irreversible death or resistant asystole which is unresponsive to twenty-minute CPR (1, 20, 31). The significant relationship of CPR duration with survival rate (62) and neurologic conditions in patients discharged after prolonged resuscitation (63, 64) causes serious ethical challenges for nurses in following medical orders for DNR, ToR, or slow code. Close monitoring of the process of CPR, discussing the outcomes of CPR in the ethics committees of hospitals, and providing CPR staff with appropriate feedback can reduce these challenges.

Most study participants noted that physicians issue DNR, early ToR, or slow code orders mostly verbally. A former study also reported the same finding (32). Another study found that one third of physicians did not feel competent enough for making appropriate ToR decisions (33). Lack of clear national guidelines for making CPR-related decisions, CPR staff's fear and concern over the legal consequences of their decisions, and ambiguities in the interpretation of CPR guidelines result in avoidance from the documentation of some CPR orders, use of futile interventions such as slow code, and wastage of healthcare resources (31–33). These consequences clearly highlight the importance of developing clear national guidelines for CPR, DNR, and ToR. A number of limitations in the present study deserve to be mentioned. First, the cross-sectional design of the current study limits our ability to form firm conclusions regarding causality. Second, in the present study, knowledge and attitude of resuscitation team nurses of two hospitals were evaluated. Therefore our finding may not be generalizable to all CPR nurses in the country or nurses in developing countries that incorporate different ethnicity, religions, cultures, and political backgrounds.

In conclusion, this study indicated that most nurses in CPR team have limited DNR and ToR knowledge and limited competence in making appropriate decisions during CPR. These issues are mainly due to the lack of clear clinical guidelines for CPR as well as CPR staff's fear over the legal consequences of DNR and ToR. These findings highlight the importance of developing a clear culturally appropriate guidelines regarding DNR and ToR, in order to prevent any potential confusion, legal or psychosocial issues and concerns for CPR nurses and improve their involvement in CPR decision making process.
